# Influence of mould growth and outdoor exposure on the efficacy of attractive targeted sugar baits against *Anopheles arabiensis* in western Kenya

**DOI:** 10.1371/journal.pone.0315472

**Published:** 2025-07-03

**Authors:** Nick O. Yalla, Jackline Kosgei, Frank Mechan, Daniel P. McDermott, Brian Polo, Seline Omondi, Elizabeth Omukunda, Eric Ochomo

**Affiliations:** 1 Centre for Global Health Research, Kenya Medical Research Institute, Kisumu, Kenya; 2 Department of Biological Sciences, Masinde Muliro University of Science and Technology, Kakamega, Kenya; 3 Innovation to Impact (I2I), Liverpool School of Tropical Medicine, Liverpool, United Kingdom; 4 Department of Clinical Sciences, Liverpool School of Tropical Medicine, Liverpool, United Kingdom; Clinton Health Access Initiative, UNITED STATES OF AMERICA

## Abstract

**Introduction:**

Attractive Targeted Sugar Baits (ATSBs) effectively target *Anopheles* mosquitoes in semi-arid, low-humidity climates. However, high humidity encourages mould growth on ATSB surfaces, and its effect on the efficacy of ATSBs against malaria vectors is yet to be determined.

This study explored how mould growth affects the performance of ATSB version 1.2 by comparing mouldy stations from exposed environments to non-mouldy stations from protected settings through laboratory bioassays on the local malaria vector, *Anopheles arabiensis*.

**Methods:**

One hundred ATSB stations were deployed in Asembo, Rarieda-Subcounty, Siaya County Six samples, consisting of three mouldy from exposed locations and three non-mouldy from protected locations were collected monthly for laboratory bioassays. These were tested alongside three new laboratory-kept ATSBs and two negative controls (water only and 77% sugar solution with water) to assess mosquito feeding and mortality over 48 hours.

**Results:**

This study found that after 12 months of outdoor exposure, the mouldiest ATSBs from exposed locations showed a non-significant reduction in *Anopheles arabiensis* feeding rates compared to the least mouldy ATSBs from protected locations 57.42% (95% CI: 45.64–68.85) vs. 74.40% (95% CI: 64.56–82.50), (P = 0.062) respectively. Mosquito mortality significantly declined on mouldy ATSBs compared to laboratory controls 95.35% (95% CI: 92.23–97.48) vs. 98.70% (95% CI: 97.87–99.30), (P = 0.002) respectively. In contrast, protected (non-mouldy) ATSBs showed only a slight reduction in mortality compared to controls 95.94% (95% CI: 90.42–97.46) vs. 98.91% (95% CI: 97.67–99.60) respectively (P = 0.009).

**Conclusion:**

This study provides evidence that environmental exposure post-deployment slightly reduced the efficacy of ATSBs in controlling *Anopheles arabiensis*, particularly beyond the recommended 6-month period. Although mould may have contributed to this reduction over 12 months, no significant difference was found between mouldy and non-mouldy ATSBs. However, mould invasion and community concerns highlight the need to replace mouldy stations to maintain effectiveness and safety.

## Background

Attractive Targeted Sugar Bait (ATSB) is a novel outdoor tool that exploits mosquito sugar-feeding behaviour to control them [1]. The concept of ATSB allows a range of insecticides or toxicants to be delivered to the mosquitoes through ingestion [2]. This has led to explorations with multiple insecticides in mosquito control. Chemical classes of insecticides that have already been tested as ATSBs include pyrethroids, carbamates, organophosphates, ivermectins, borates, neonicotinoids, spinosyns, pyriproxyfen, pyrroles, double-stranded RNA (dsRNA), biopesticides and phenylprazoles [2–10].

Previously, ATSB was implemented by spraying non-flowering vegetation close to mosquito larval habitats, targeting the newly emerged sugar-seeking vectors [11]. This approach notably decimated mosquito populations of different genera in varied study regions [12,13] but could be environmentally unsustainable because of the potentially hazardous impact on non-target organisms (NTOs) [10].

The version 1.2 ATSB stations used in this experiment contained fruit syrup as an attractant and stimulant for inducing the mosquitoes to feed and ingest a lethal dose of dinotefuran, a neonicotinoid insecticide, and were manufactured by Westham Co. Hod-Hasharon, Israel. They also contained uranine as a fluorescent tracer. The ATSB is designed to be hung outdoors on a wall under a roof to control mosquitoes in the peridomestic space [14]. A previous version of this ATSB station was tested in Mali and achieved a significant density reduction of 90% of female *Anopheles gambiae s.l*. past their second gonotrophic cycle [15].

When the new version 1.2 of the same ATSB product was deployed in western Kenya for a large-scale epidemiological trial, mould growth was observed on the many bait station membranes. This mould growth was observed through regular monitoring to be widespread on structures with short roof lengths overhanging the walls where the ATSBs were mounted, so the products were exposed to weather elements like sun and rainfall [14]. In Zambia, mould growth contributed to approximately 28% of all ATSB damage, and it was closely linked to the duration of deployment [16]. ATSBs that sustained tears were particularly vulnerable to mould attacks, especially when left in the field for long periods without replacement. Furthermore, the characteristics of the deployment site, for example whether the ATSB was protected from rainfall or not, also played a role in predicting the likelihood of mould damage [16].

Most moulds favourably proliferate on paper products, cardboard ceiling tiles, wood products, insulation materials, upholstery, and other fabrics, breaking them down [17]. The ideal growth conditions for these moulds are moisture availability, carbohydrates like sucrose, and sufficient oxygen [18]. Low diurnal temperature ranges and high humidity characterize the western Kenya region, particularly during the long rainy season. Moreover, ATSB is approximately 70% sugar solution, a carbohydrate readily broken down by the mould. This provides favourable conditions for mould growth on the bait station membranes that might be expected to reduce the efficacy of ATSBs in controlling malaria vectors by either lowering their attractiveness to or feeding rates on the bait stations by the target vectors [18,19]. Some moulds are toxic, while others have been useful to humans in different ways [19]. There has been no reported work that has aimed to characterise the mould species that grow on the ATSBs in the areas where they have been deployed. The current policy is to remove the bait stations on walls if they become mouldy with mould spots bigger than a pencil eraser with a diameter of approximately 0.5 inches [14]. This replacement criterion can increase wastage and pose a challenge for intervention coverage when there isn’t a rigorous monitoring and replacement programme. Additionally, there are concerns about the impact of mould growth on people’s health.

There is currently no documented evidence of the impact of mould growth on ATSB’s ability to influence mosquito feeding and mortality rates. This study examined the influence of mould growth on ATSB efficacy by comparing the feeding rates of malaria vectors on mouldy versus non-mouldy ATSBs in laboratory bioassays. In addition, this study evaluated the effectiveness of the ATSBs beyond the six months recommended by the manufacturer to assess the persistence of the active ingredient. The results of this study provided insights into whether the natural presence of the mould can impact the efficacy of ATSB as a complementary outdoor malaria vector control strategy in western Kenya.

## Methods

### Study site and design

One hundred uranine-dye stained ATSBs were hung in Asembo, Siaya County, Kenya which was part of the study site for the large-scale, cluster-randomized controlled trial with ATSBs implemented between March 2022 and March 2024 [14]. Fifty ATSBs were hung on a structure wall without a roof overhang, termed herein as (an exposed location), and another fifty were hung on a separate wall with a roof overhang, termed herein as (a protected location), protecting the bait stations from direct effects of weather elements such as rain, sunlight, and wind. The ATSBs were hung on the walls 1.8 meters from the ground.

### Mould culturing and morphological identification

To investigate and isolate the mould growing on ATSB stations, six bait stations that exhibited significant mould growth and were more than six months old were randomly selected from Asembo and transported to the laboratory for culturing. A pair of forceps was sterilized by dipping in 70% ethanol and subsequently briefly flamed over a Bunsen burner. The sterile forceps was used to gently scrape off a small portion of mould from the ATSB surface, ensuring that both mouldy and adjacent areas were included to capture all species of fungi growing. Following this, 4.8 grams of potato dextrose agar (PDA) was weighed and transferred to a sterile conical flask. The PDA was then suspended in 120 ml of distilled water, stirred for 1 minute using a magnetic stirrer, and heated on a hot plate until the medium was completely dissolved. The medium was autoclaved for 20 minutes at 121°C and 15 psi. After autoclaving, the media was allowed to cool to about 45ºC, permitting the gel to solidify. Ten percent tartaric acid was added to the medium to lower the pH to approximately 3.5 to inhibit bacterial growth. Subsequently, 20 ml of the medium was dispensed into six petri dishes each. The spread plate streaking technique was applied to isolate the mould inoculum, picked from six different ATSBs, onto each petri dish. The plates were then incubated for 7 days at 27ºC [20]. Finally, the colony was observed under a light microscope (Leica DM750M, Suzhou, China) to reveal the fungal morphology.

### Mosquito samples

*Anopheles* mosquitoes used in the tests were collected as larvae from Ahero (0.16°S, 35.0°E) in Kisumu County, Kenya, using the larval dipping method. The larval sampling was conducted for two weeks each month from January to September 2023. The field-collected larvae were raised under laboratory conditions with temperature maintained at 28 ± 2 ºC and a relative humidity of 80 ± 10%. The larvae were fed on TetraMin fish food up to the pupal stage, after which they were transferred to plastic cups using 3 ml Pasteur pipettes. The cups containing pupae were placed in (30x30x30) cm cages for emergence. Adults were maintained on a 10% sugar solution soaked in cotton wool until 3–5 days post-emergence, before used for bioassays based on the WHO recommendation for insecticide resistance monitoring [21].

### Mosquito-ATSB feeding rates and mortality assessment

**Fig 1:**
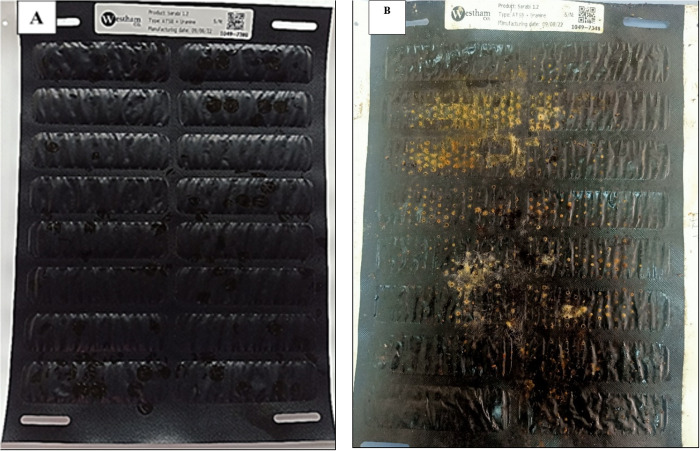
Pictures of new ATSB freshly removed from packaging laboratory kept (A) and field-collected mouldy ATSBs (B).

Field ATSBs were selected monthly for bioefficacy tests in the KEMRI laboratory between October 2022 and October 2023, a time span longer than the manufacturer’s prescribed period of six months [14]. Every month, six ATSBs, three non-mouldy from the protected and three mouldy from the exposed locations, were removed and transported safely in portrait orientation in wooden transportation boxes of dimensions 35 cm x 25 cm x 7 cm to the KEMRI laboratory in Kisumu County for bioassays. The most mouldy ATSBs from the exposed location and non-mouldy from the protected location were scouted and selected for bioassay. ATSBs selected for bioassay were selected if more than half of the bait station membrane had mould covering their surfaces. The protected location had a roof overhang, which provided protection to the ATSBs from direct effects of weather elements. In addition, three laboratory-kept ATSBs that had never been used were tested alongside the field bait stations each month to assess *Anopheles* mosquito feeding rates in the laboratory. The laboratory kept ATSBs were used as positive controls to the field collected bait stations. The laboratory kept ATSBs were from the same manufacturing batch as the field collected ones and were freshly opened from their packaging materials at the start of the experiment. The laboratory ATSB storage condition was 60% relative humidity and temperature of 25°C. The field-collected most mouldy, non-mouldy, and laboratory-kept ATSBs were tested for mosquito feeding rates in cages with access to *ad libitum* water soaked in cotton wool. In addition, negative controls of water alone and water with 77% sugar solution (considered similar to ATSB in sugar concentration) were also set alongside the cages with ATSBs. This study did not collect field weather data but the temperature ranges between 14–36°C while humidity ranges between 60–70% [22] (Fig 1).

All the test cages were supplied with *ad libitum* water including the 77% sugar cage.

Mosquito bioassays were conducted in plastic Bugdorm cages measuring (30x30x30 cm.) Each cage was fitted with one ATSB, positioned vertically on one of the four walls of the cage frame, and firmly secured with masking tape. Including the controls, cohorts of 3–5 day-old, one hundred and twenty Anopheles mosquitoes (60 males and 60 females) were released into each of the experimental cages. The choice of 3–5 day old mosquitoes was based on WHO recommendations for insecticide resistance testing [21]. Nine hundred and sixty mosquitoes were used per month for the bioassays. Mosquito samples used were always starved 24 hours prior to the experiment before being released into the cages containing the bait stations. The mosquitoes were allowed to feed on the bait stations for 48 hours, starting in the early afternoon at 1500 hours. At the 24-hour time point, all dead male and female mosquitoes in the treatment and control cages were aspirated out of the cages and placed in specimen vials. The remaining live mosquitoes were left in the cages with the bait station and monitored for a further 24 hours. At the end of 48 hours, all dead and alive mosquitoes in all treatments and control cages were aspirated out. The live mosquitoes were killed by freezing in a −20^0^C freezer. All the samples, including those sampled at 24 hours, were examined for uranine dye to detect if they had fed on the ATSB. This was done by scanning through the mosquito abdomens with a fluorescent microscope (Model: Leica Mz 10f,10 Parkway North Blvd, Suite 300, Deerfield, Illinois 60015 United States). The bio-assayed mosquitoes were individually placed onto the fluorescent microscope stage in a Petri dish directly under the field of view to assess the presence of uranine dye. White light and a green UV filter was used for microscopic illumination and for visualizing uranine fluorescence, respectively. An observation of a light green colour of uranine dye in the mosquito’s abdomen/gut indicated feeding, while the absence of the dye colour meant the mosquito did not feed. PCR analyzed two hundred and four of the mosquitoes for species identification.

### Statistical analysis

Generalized Linear Mixed Models (GLMMs) in R statistical software analyzed associations between station location, time-point, and outcomes; proportion fed (dye-positive) and proportion dead (% mortality). A random effect term was included to account for unexplained variation between assays identified by the ID of each bait station. ATSB location was treated as a categorical variable, while the time-point was considered a continuous variable. The statistical significance of each parameter, including an interaction term between station location and time-point, was evaluated using log-likelihood ratio tests (LRTs), comparing models with and without the parameter. Post hoc analysis was conducted using the ‘lsmeans’ lme4, and lmtest, packages in R version 4.4.1.

### Ethical considerations

The study was approved by the Kenya Medical Research Institute/Scientific and Ethics Review Unit (KEMRI/SERU), number (SERU 3613). It was also approved by the Institutional Review Board of the US Centres for Disease Control and Prevention (#7112) and the Liverpool School of Tropical Medicine Research Ethics Committee (18-015) in agreement with KEMRI SERU.

## Results

### Mould culture identification

**Fig 2:**
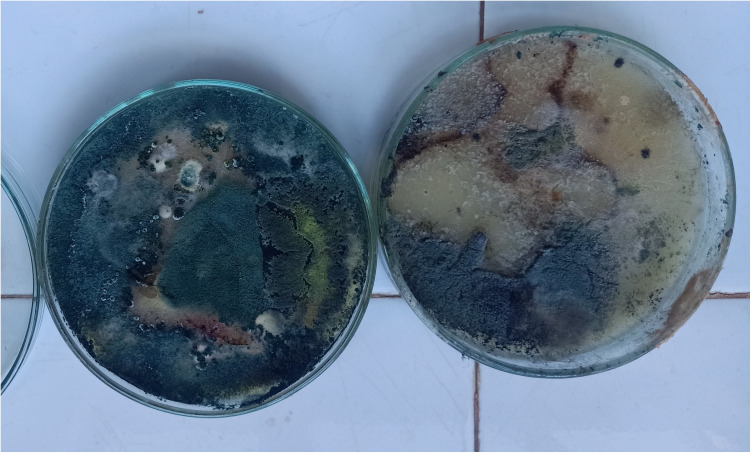
Picture of mould colony cultured on potato dextrose agar on day seven.

The PDA culture characteristics of the mould revealed a mixed colony of fungal species that initially appeared cottony white after two days of incubation but turned black in larger parts of the petri dishes after seven days, producing conidial spores. By the end of the incubation period, most of the culture remained black. The fungal culture was examined under a light microscope (Leica DM750M, Suzhou, China). *Aspergillus niger* mould was identified to be the dominant species covering 99% surfaces of the culture medium in all the plates assessed (Fig 2).

### Mosquito samples

Ninety-five percent of the 204 Anopheles mosquitoes tested were confirmed to be *Anopheles arabiensis* by PCR, while ten samples did not amplify. Whole samples were used for DNA extraction using an ethanol precipitation method [23]. Polymerase chain reaction (PCR) was used to distinguish between the sibling species of the *An. gambiae* sl*.* species complex [24]. Selection of the two hundred and four mosquitoes for PCR identification was guided by the entomological surveillance studies supporting a larger evaluation of epidemiological efficacy of ATSB [25]. This study specified that a maximum of two hundred mosquitoes collected in 16 of the 70 clusters in a small geographical area were sufficient for PCR identification.

### Cumulative Mosquito feeding rates at 48 hours

**Fig 3:**
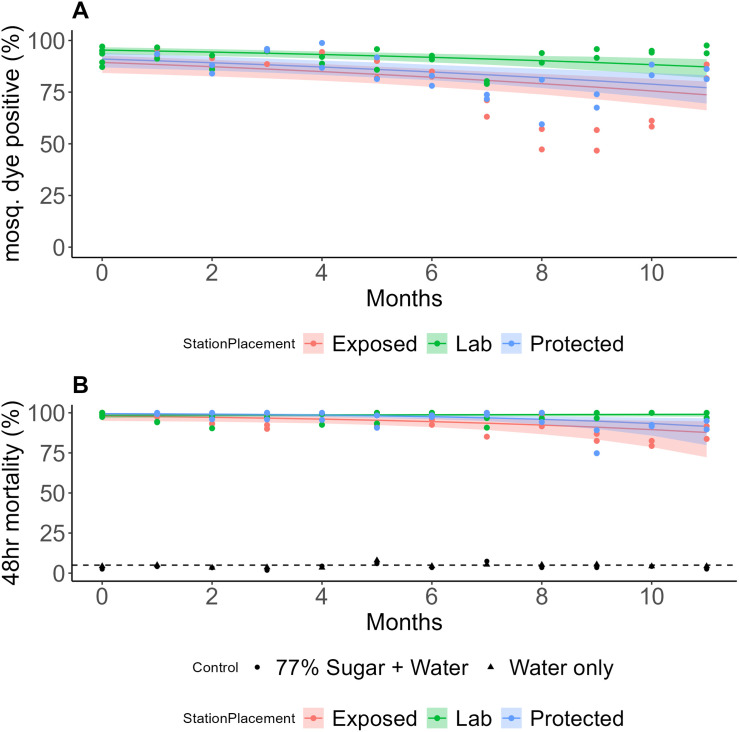
Mosquito dye positive and 48-hour mortality rates (A and B) on ATSBs, respectively. The coloured bands represent 95% intervals.

Time and bait station location (“exposed” mouldy vs. “protected” non-mouldy) were significant predictors of the cumulative proportion of mosquitoes showing dye-positive results at 48 hours (P* *< 0.001). A significant interaction between the time-point and bait station location (P < 0.001) was observed (Fig 3A). By 5 months, the ATSBs in the field had a significantly lower mosquito feeding rate compared to the laboratory control, with rates of 87.82% (95% CI: 84.12–91.09) and 92.63% (95% CI: 90.81–94.17) respectively (P = 0.001). ATSBs sampled from the protected location exhibited significantly lower dye-positivity rates compared to the lab-kept ATSBs at month five, with rates of 86.34% (95% CI: 82.78–89.11) and 92.63% (95% CI: 90.81–94.17) respectively (P < 0.001). This study found no significant difference in feeding rates between exposed (mouldy) and protected (non-mouldy) stations at any time point. However, at the final time point, mouldy stations had lower feeding rates (57.42%, 95% CI: 45.64–68.85) compared to non-mouldy stations (74.40%, 95% CI: 64.56–82.50) (P = 0.062).

### Cumulative mosquito mortality rates at 48 hours

Time and bait station location were found to be significant predictors of the cumulative proportion of mosquito mortality at 48 hours (P = 0.009 and P = 0.001, respectively). A significant interaction between the time-point and bait Station location (P < 0.001) was observed (Fig 3B). The exposed location (Mouldy) had a significant reduction in mosquito mortality compared to laboratory control by month five with rates of 95.35% (95% CI: 92.23–97.48) and 98.70% (95% CI: 97.87–99.30) respectively (P = 0.002). The mortality estimated dropped in month eight, which would raise concerns about its use beyond this period. The protected location experienced a small reduction in mortality by the seventh month compared to the laboratory controls, 95.94% (95% CI: 90.42–97.46) vs. 98.91% (95% CI: 97.67–99.60) respectively (P = 0.009), though the mortality rate remained above 95% until the eighth month. There were no significant differences between the exposed and protected ATSBs at any time-point, with mean 48-hour mortality rates at the final time-point being 87.76% (95% CI: 74.17–94.37) vs. 91.54% (95% CI: 81.48–96.83) respectively (P = 0.807). Less than 10% and 5% cumulative mortalities were recorded in water only and 77% sugar solution plus water controls, respectively.

## Discussion

This study found that mouldy ATSBs maintained similar feeding rates for up to five months post-deployment in both laboratory and field conditions (>85% in outdoor settings). While no significant difference was observed between exposed (mouldy) and protected (non-mouldy) ATSBs at specific time points, exposed stations showed lower overall feeding rates, with a notable decline after eight months. Exposed and non-exposed ATSBs caused similar mosquito mortality compared to new lab-kept ATSBs. The reduced late-cycle feeding rate warrants further investigation as it may impact product efficacy. The presence of mould or weather factors (humidity and temperature) specific to exposed versus protected environments appears to affect mosquitoes’ ability to feed on ATSBs. Mould may block ATSB pores or harden membrane surfaces, making it difficult for mosquitoes to probe and ingest the toxin. This suggests that substantial mould growth on the ATSBs may warrant the removal or replacement of the product from the walls if better mosquito control with ATSB is to be attained in environments favouring fungal growth.

Culturing revealed different fungal species growing on the product. This study identified *Aspergillus niger* as the most dominant mould in the colony but, unfortunately, failed to characterize other mould species that formed part of the culture. Given the small samples of mouldy ATSBs assayed, this study might not have involved all the types of mould growing on the product in the entire study area, and further characterisation studies are needed. The safety level of most of these moulds that grow on the product is still unknown. Thus it remains a concern that despite not detecting a major problem for efficacy in laboratory feeding and mortality tests, the risk of exposure to these moulds needs further investigation. Mould damage to ATSB stations seems to increase with longer deployment durations, showing a direct relationship between the prevalence of mould and the extent of tear damage [16]. The findings demonstrate that, despite mould growth, the stations effectively delivered a toxic dose of the active ingredient up to the twelfth month. However, extending deployment beyond the current six-month period without further station optimization raises concerns. Rapid identification and replacement of torn ATSBs may therefore reduce the rate of development of mould but this might require high bait station turnover. However, frequent replacement of the ATSBs is not sustainable and might make deployment very expensive. Mould and other types of damage, may also influence community acceptance, making it important to balance operational feasibility with community perceptions for successful deployment [16]. *Aspergillus niger*, found to be the most dominant mould in this study, is, however, not considered harmful to humans in lower doses, with between 200–500 spores typically not an issue and staying within the normal range. Moreover, when *Penicillium, Aspergillus, or Cladosporium* levels range between 500–1500 spores, remediation is typically not required as they are regarded not harmful to human [26–28]. Most people potentially breathe in *Aspergillus* spores every day without getting sick, as *Aspergillus niger* is generally regarded as a non-pathogenic fungus widely distributed in nature [18,19,29]. Only in a few cases has *Aspergillus niger* has been able to colonize the human body as an opportunistic invader, and in almost all these cases, the patients have a history of severe illness or immunosuppressive treatment [30]. However, the authors recognise the need for a more rigorous screening strategy to identify if any mould that may cause pathology in household members who are in close proximity to the ATSB can be found. Even in the absence of efficacy and health concerns, the community acceptability of an intervention that grows mould on people’s homes may present an issue.

Given these concerns about ATSB, there is a need to alter the design or increase the anti-mould agents in the product to reduce the rate of mould development. It is essential to note that weather conditions, particularly humidity, play a significant role in the propagation of mould in addition to moisture, carbohydrate substrate, and moderately low-temperature conditions [16]. Western Kenya provides conducive conditions to mould growth compared to Mali, which is drier for the better part of the year. This probably explains the lack of mould occurrence in Mali during the testing of ATSBs there as opposed to the current setting [15]. Further, this difference in climate highlights the importance of considering environmental factors when assessing the efficacy of vector control tools, especially those aimed to be deployed for outdoor use. More research is needed on how mould on ATSB impedes mosquito feeding. Whether it blocks or hardens the bait station membrane making it impossible for the mosquito proboscis to pierce and suck the bait solution, is unknown. In the natural setting, mould might significantly lower the product efficacy through reduced feeding rates due to blocked pores or hardened membrane surfaces of the ATSBs. The higher reduction in mosquito feeding rates relative to mosquito mortality observed in this study is probably due to the drying of the active ingredient on ATSB membrane surfaces making it difficult for mosquitoes to ingest sufficient volumes of the bait. Future studies should consider collecting weather data to evaluate the dynamics of mosquito feeding rates as well as mould growth, with consideration of temperature, rainfall, and humidity, providing a more comprehensive understanding of the impact of weather variables on mould growth and the product’s effectiveness. This feeding assay was conducted in cages, which may not fully replicate mosquito behaviour in natural settings. With only one hundred ATSBs used and only 3 mouldy and 3 non-mouldy ATSBs compared at each time point, the study suggests that future research should include a larger sample size to provide more conclusive insights into the impact of mould on ATSB performance. Worth noting is the fact that bait station location was confounded with mould presence, making it impossible to distinguish these factors in this study. Future studies should consider using ATSBs from the same environment.

## Conclusion

This study demonstrated that environmental exposure of the ATSB station in western Kenya caused a gradual decline in its effectiveness against *Anopheles arabiensis* mosquitoes, with decreasing feeding and mortality rates during laboratory bioassays, especially beyond the recommended 6-month deployment period. While there was evidence that the presence of mould exacerbated this effect over a 12-month outdoor exposure period, there was no significant efficacy difference at any one-time point between the field-collected mouldy and non-mouldy ATSBs. Owing to the overall reduction observed, the absence of safety data around the moulds that are present, and community perceptions, we still recommend the removal and replacement of any ATSB stations that become torn or mouldy.

## Supporting information

S1 Study Data Data. (XLSX)

## References

[1] N’Guessan R, Camara S, Rowland M, Ahoua Alou LP, Wolie RZ, Zoh MG, et al. Attractive targeted sugar bait: the pyrrole insecticide chlorfenapyr and the anti-malarial pharmaceutical artemether-lumefantrine arrest Plasmodium falciparum development inside wild pyrethroid-resistant Anopheles gambiae s.s. mosquitoes. Malar J. 2023;22(1):344. doi: 10.1186/s12936-023-04758-1 37946208 PMC10636997

[2] Fiorenzano JM, Koehler PG, Xue R-D. Attractive Toxic Sugar Bait (ATSB) For Control of Mosquitoes and Its Impact on Non-Target Organisms: A Review. Int J Environ Res Public Health. 2017;14(4):398. doi: 10.3390/ijerph14040398 28394284 PMC5409599

[3] N’Guessan R, Camara S, Rowland M, Ahoua Alou LP, Wolie RZ, Zoh MG, et al. Attractive targeted sugar bait: the pyrrole insecticide chlorfenapyr and the anti-malarial pharmaceutical artemether-lumefantrine arrest Plasmodium falciparum development inside wild pyrethroid-resistant Anopheles gambiae s.s. mosquitoes. Malar J. 2023;22(1):344. doi: 10.1186/s12936-023-04758-1 37946208 PMC10636997

[4] Xue R-D, Kline DL, Ali A, Barnard DR. Application of boric acid baits to plant foliage for adult mosquito control. J Am Mosq Control Assoc. 2006;22(3):497–500. doi: 10.2987/8756-971X(2006)22[497:AOBABT]2.0.CO;2 17067052

[5] Qualls WA, Müller GC, Traore SF, Traore MM, Arheart KL, Doumbia S, et al. Indoor use of attractive toxic sugar bait (ATSB) to effectively control malaria vectors in Mali, West Africa. Malar J. 2015;14:301. doi: 10.1186/s12936-015-0819-8 26242186 PMC4524285

[6] Qualls WA, Xue R, Revay EE, Allan SA, Müller GC. Implications for operational control of adult mosquito production in cisterns and wells in St. Augustine, FL using attractive sugar baits. Acta Trop. 2012;124(2):158–61. 22820024 10.1016/j.actatropica.2012.07.004

[7] Xue R-D, Ali A, Kline DL, Barnard DR. Field evaluation of boric acid- and fipronil-based bait stations against adult mosquitoes. J Am Mosq Control Assoc. 2008;24(3):415–8. doi: 10.2987/5683.1 18939695

[8] Revay EE, Müller GC, Qualls WA, Kline DL, Naranjo DP, Arheart KL, et al. Control of Aedes albopictus with attractive toxic sugar baits (ATSB) and potential impact on non-target organisms in St. Augustine, Florida. Parasitol Res. 2014;113(1):73–9. doi: 10.1007/s00436-013-3628-4 24122115 PMC3945672

[9] Beier JC, Müller GC, Gu W, Arheart KL, Schlein Y. Attractive toxic sugar bait (ATSB) methods decimate populations of Anopheles malaria vectors in arid environments regardless of the local availability of favoured sugar-source blossoms. Malar J. 2012;11:31. doi: 10.1186/1475-2875-11-31 22297155 PMC3293779

[10] Khallaayoune K, Qualls WA, Revay EE, Allan SA, Arheart KL, Kravchenko VD, et al. Attractive toxic sugar baits: control of mosquitoes with the low-risk active ingredient dinotefuran and potential impacts on nontarget organisms in Morocco. Environ Entomol. 2013;42(5):1040–5. doi: 10.1603/EN13119 24331613 PMC3918905

[11] Revay EE, Schlein Y, Tsabari O, Kravchenko V, Qualls W, De-Xue R, et al. Formulation of attractive toxic sugar bait (ATSB) with safe EPA-exempt substance significantly diminishes the Anopheles sergentii population in a desert oasis. Acta Trop. 2015;150:29–34. doi: 10.1016/j.actatropica.2015.06.018 26119042 PMC4720916

[12] Naranjo DP, Qualls WA, Müller GC, Samson DM, Roque D, Alimi T, et al. Evaluation of boric acid sugar baits against Aedes albopictus (Diptera: Culicidae) in tropical environments. Parasitol Res. 2013;112(4):1583–7. doi: 10.1007/s00436-013-3312-8 23388730

[13] Müller GC, Beier JC, Traore SF, Toure MB, Traore MM, Bah S, et al. Successful field trial of attractive toxic sugar bait (ATSB) plant-spraying methods against malaria vectors in the Anopheles gambiae complex in Mali, West Africa. Malar J. 2010;9:210. doi: 10.1186/1475-2875-9-210 20663142 PMC2914067

[14] Eisele TP, Kleinschmidt I, Sarrassat S, TerKuile F, Miller J, Chanda J, et al. Attractive Targeted Sugar Bait Phase III Trial Group. Attractive targeted sugar bait phase III trials in Kenya, Mali, and Zambia. Trials. 2022;23:640. doi: 10.1186/s13063-022-06555-8 35945599 PMC9361277

[15] Traore MM, Junnila A, Traore SF, Doumbia S, Revay EE, Kravchenko VD, et al. Large-scale field trial of attractive toxic sugar baits (ATSB) for the control of malaria vector mosquitoes in Mali, West Africa. Malar J. 2020;19(1):72. doi: 10.1186/s12936-020-3132-0 32059671 PMC7023716

[16] Kyomuhangi I, Yukich J, Saili K, Orange E, Masuzyo MH, Mwenya M, et al. Evaluating trends in damage to attractive targeted sugar baits (ATSBs) deployed during the second year of a two-year Phase III trial in Western Zambia. Malar J. 2024;23(1):263. doi: 10.1186/s12936-024-05089-5 39210405 PMC11363357

[17] Samson RA, Visagie CM, Houbraken J, Hong S-B, Hubka V, Klaassen CHW, et al. Phylogeny, identification and nomenclature of the genus Aspergillus. Stud Mycol. 2014;78:141–73. doi: 10.1016/j.simyco.2014.07.004 25492982 PMC4260807

[18] Martínez J, Nevado A, Suñén E, Gabriel M, Vélez-Del-Burgo A, Sánchez P, et al. The Aspergillus niger Major Allergen (Asp n 3) DNA-Specific Sequence Is a Reliable Marker to Identify Early Fungal Contamination and Postharvest Damage in Mangifera indica Fruit. Front Microbiol. 2021;12:663323. doi: 10.3389/fmicb.2021.663323 34262539 PMC8273346

[19] Mousavi B, Hedayati MT, Hedayati N, Ilkit M, Syedmousavi S. Aspergillus species in indoor environments and their possible occupational and public health hazards. Curr Med Mycol. 2016;2(1):36–42. doi: 10.18869/acadpub.cmm.2.1.36 28681011 PMC5490296

[20] McClenny N. Laboratory detection and identification of Aspergillus species by microscopic observation and culture: the traditional approach. Med Mycol. 2005;43(Suppl 1):S125-8. doi: 10.1080/13693780500052222 16110804

[21] WHO. World malaria report 2017. Geneva: World Health Organization; 2017 [cited 16 May 2018]. Available from: http://www.who.int/malaria/publications/world-malaria-report-2017/

[22] Nyawanda BO, Khagayi S, Ochomo E, Bigogo G, Kariuki S, Munga S, et al. The influence of malaria control interventions and climate variability on changes in the geographical distribution of parasite prevalence in Kenya between 2015 and 2020. Int J Health Geogr. 2024;23(1):22. doi: 10.1186/s12942-024-00381-8 39465413 PMC11514743

[23] Coetzee M. Key to the females of Afrotropical Anopheles mosquitoes. 2020. 10.1186/s12936-020-3144-9 PMC7020601 32054502

[24] Müller P, Pflüger V, Wittwer M, Ziegler D, Chandre F, Simard F, et al. Identification of cryptic Anopheles mosquito species by molecular protein profiling. PLoS One. 2013;8(2):e57486. doi: 10.1371/journal.pone.0057486 23469000 PMC3585343

[25] Ogwang C, Samuels AARON, McDermott DANIELP, Kamau A, Lesosky M, Obiet K, et al. Attractive targeted sugar baits for malaria control in western Kenya (ATSB-Kenya) – Effect of ATSBs on epidemiologic and entomologic indicators: a phase III, open-label, cluster-randomised, controlled trial. 10.1371/journal.pgph.0004230 PMC12200848 40570028

[26] Sweis IE, Cressey BC. Potential role of the common food additive manufactured citric acid in eliciting significant inflammatory reactions contributing to serious disease states: A series of four case reports. Toxicol Rep. 2018;5:808–12. doi: 10.1016/j.toxrep.2018.08.002 30128297 PMC6097542

[27] Weber DJ. Preventing healthcare-associated Aspergillus infections. Medical Mycology. 2009;47. 10.1080/13693780802709073 19274596

[28] EPA. Environmental Protection Agency Final Risk Assessment for Aspergillus niger. 1997.

[29] Rhodes JC. Aspergillus fumigatus: growth and virulence. Med Mycol. 2006;44 Suppl 1:S77-81. doi: 10.1080/13693780600779419 17050423

[30] Schuster E, Dunn-Coleman N, Frisvad JC, Van Dijck PWM. On the safety of Aspergillus niger--a review. Appl Microbiol Biotechnol. 2002;59(4–5):426–35. doi: 10.1007/s00253-002-1032-6 12172605

